# Supplementary material for: Mechanisms involved in suppression of osteoclast supportive activity by transforming growth factor-β1 via the ubiquitin-proteasome system
Source: PLoS One. 2022 Feb 23;17(2):e0262612. doi: 10.1371/journal.pone.0262612 (PMC8865688; doi:10.1371/journal.pone.0262612)
Supplement: S1 Raw images — (PDF) [file pone.0262612.s001.pdf]

Figure.1D

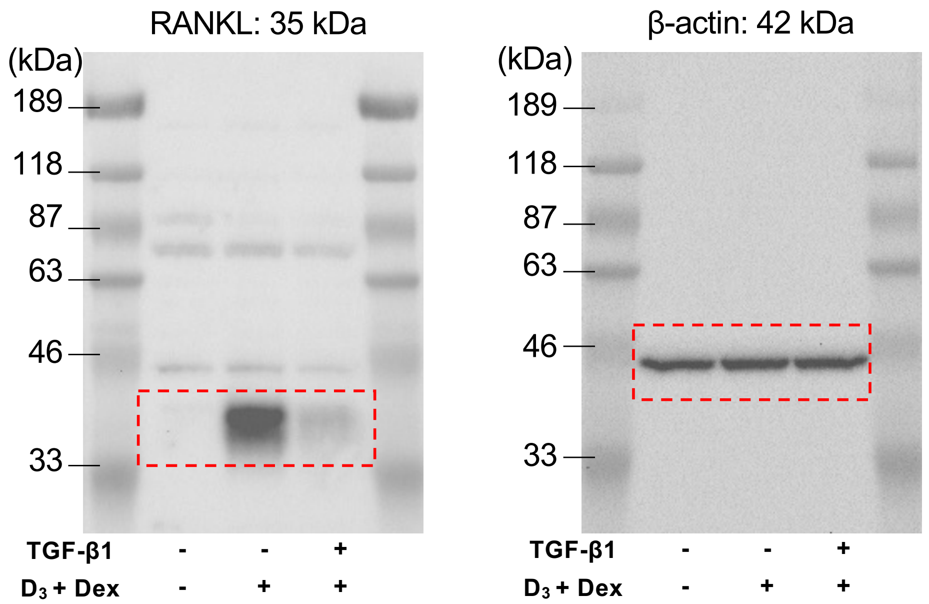

Figure.3B

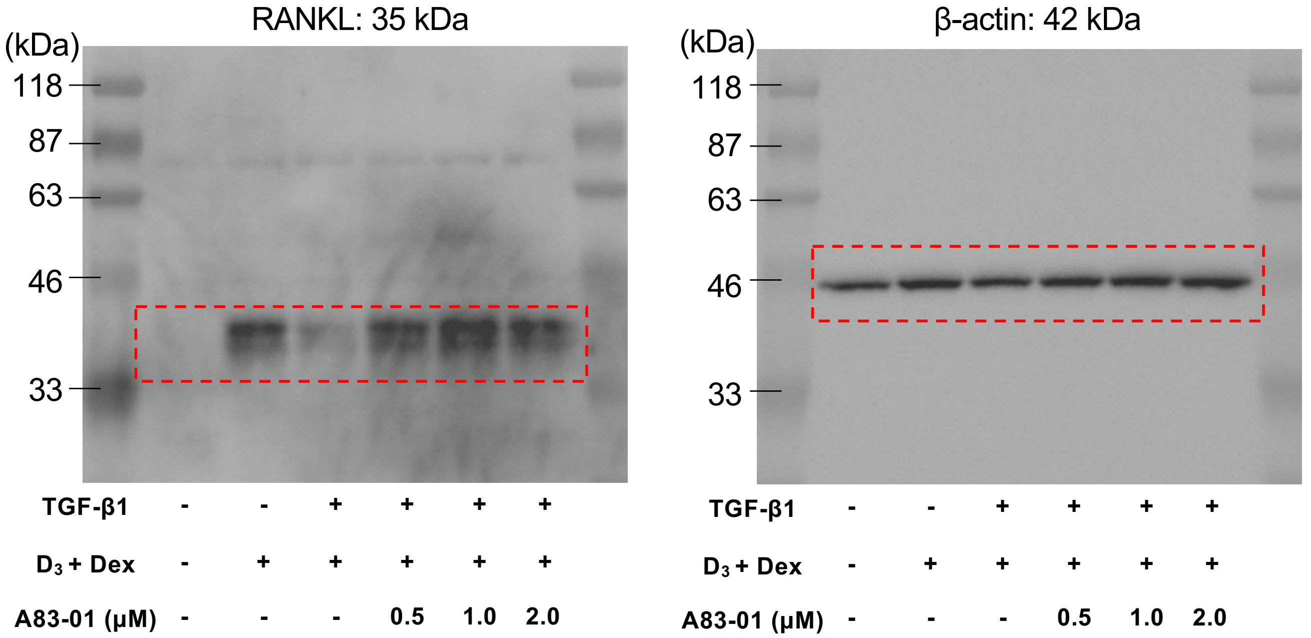

Image LabTM® 2.0 software (Bio-Rad, Hercules, CA, USA)

Figure.4A

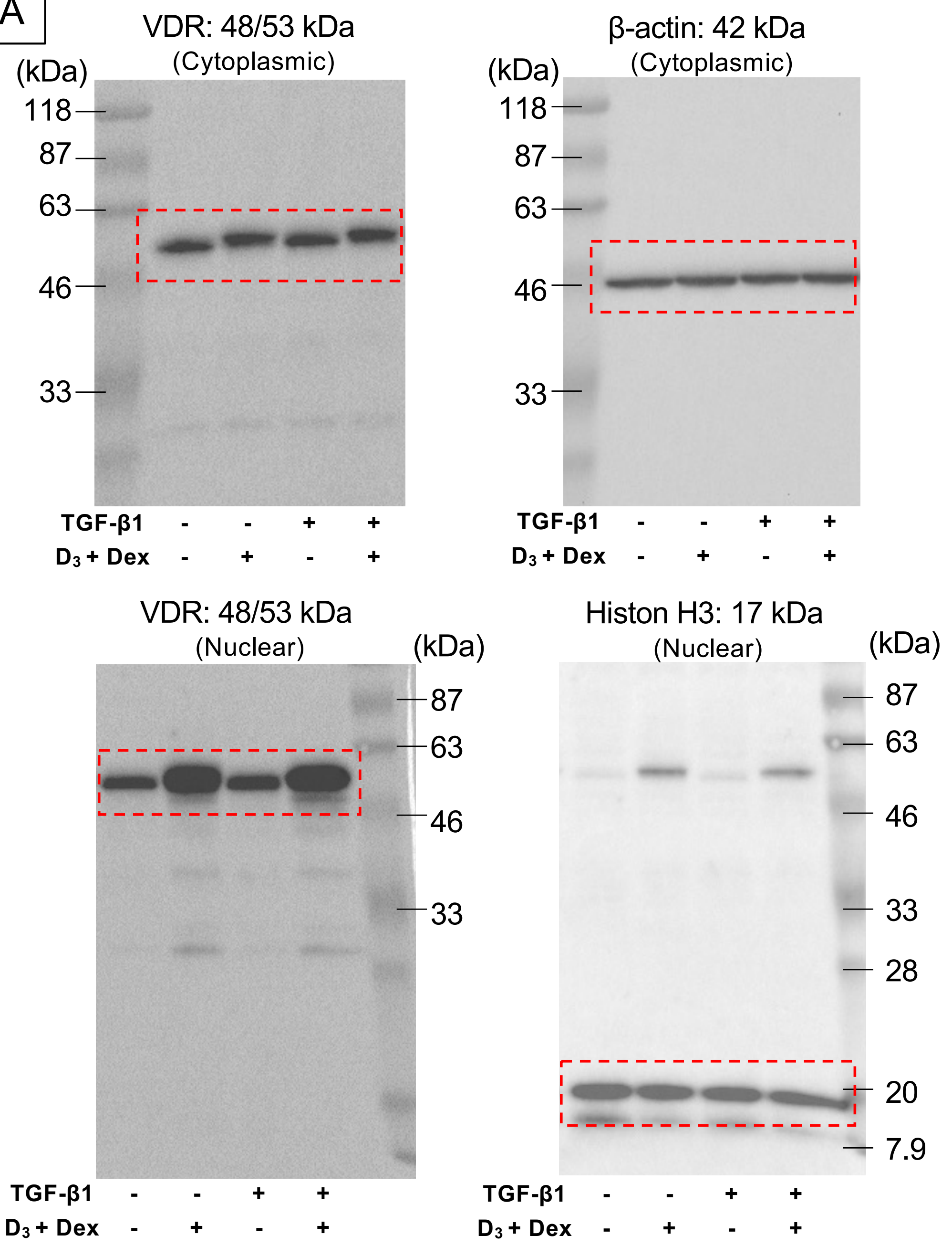

Figure.4B

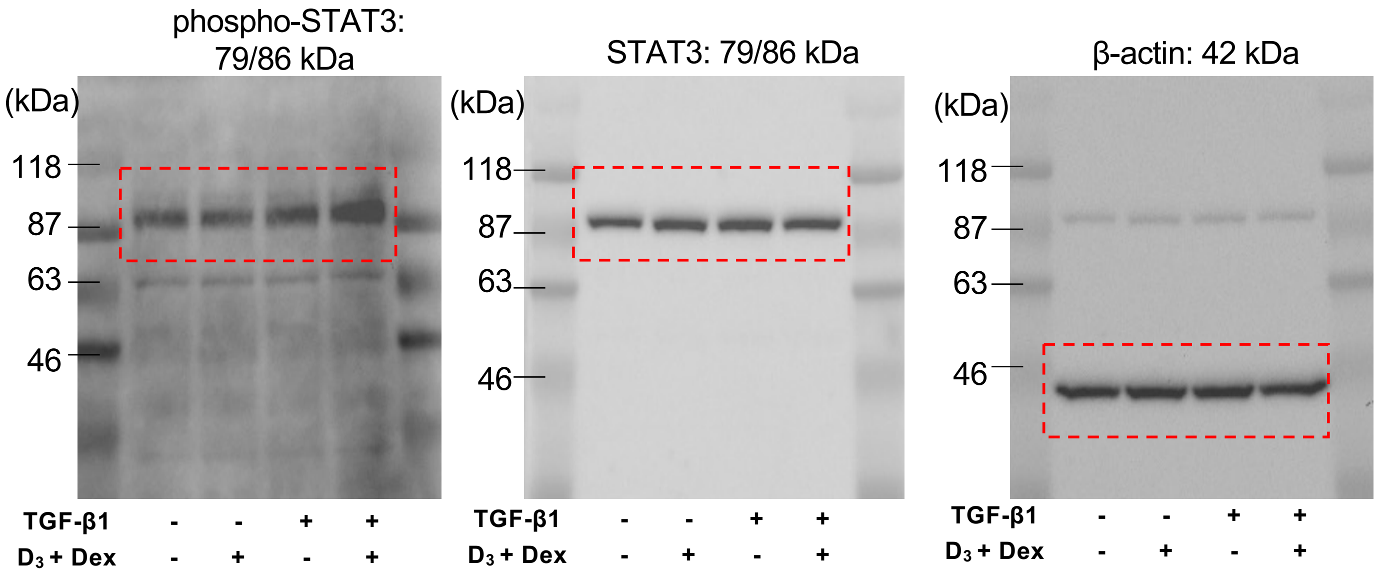

Image LabTM® 2.0 software (Bio-Rad, Hercules, CA, USA)

Figure5A

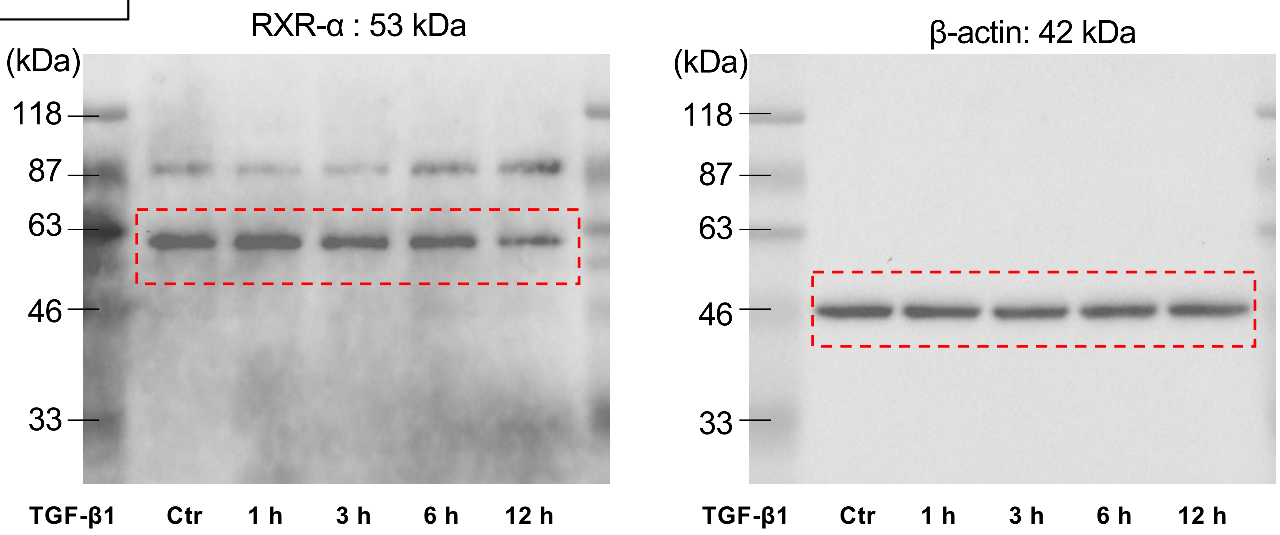

Figure.5C

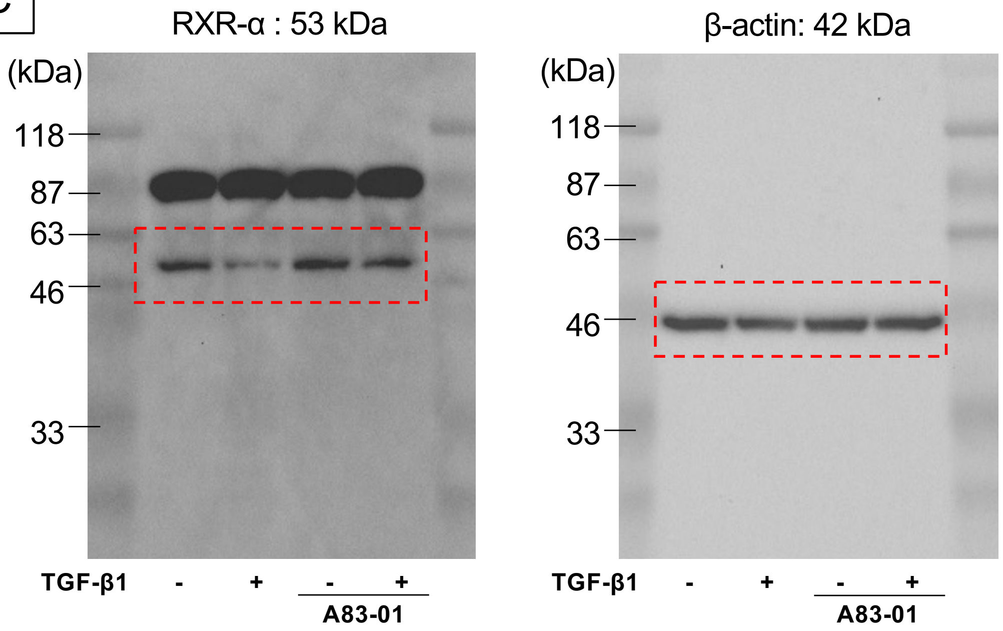

Figure.5D

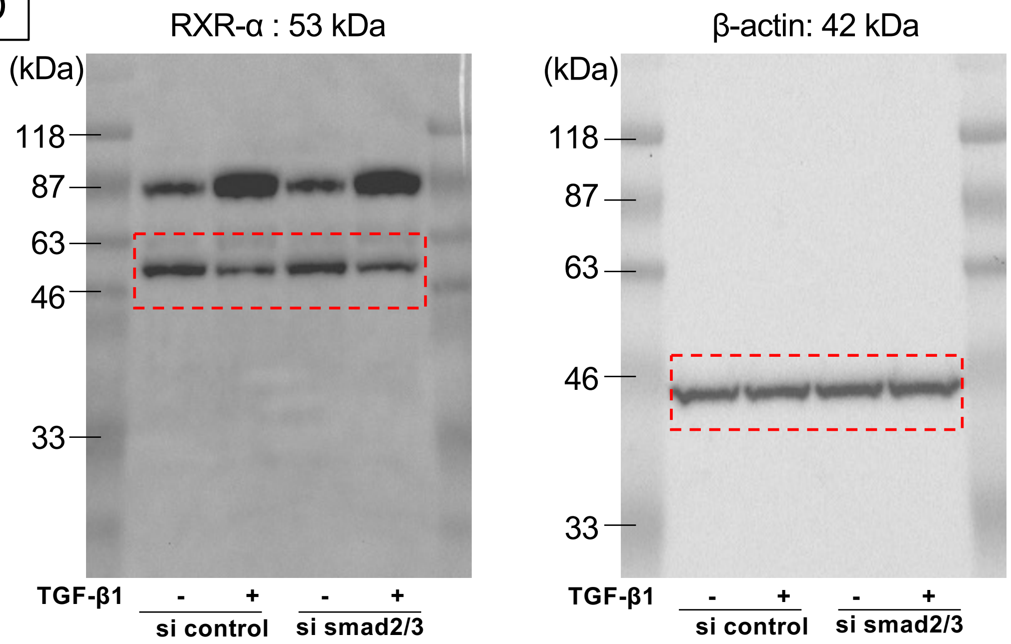

Image LabTM® 2.0 software (Bio-Rad, Hercules, CA, USA)

Figure.6A

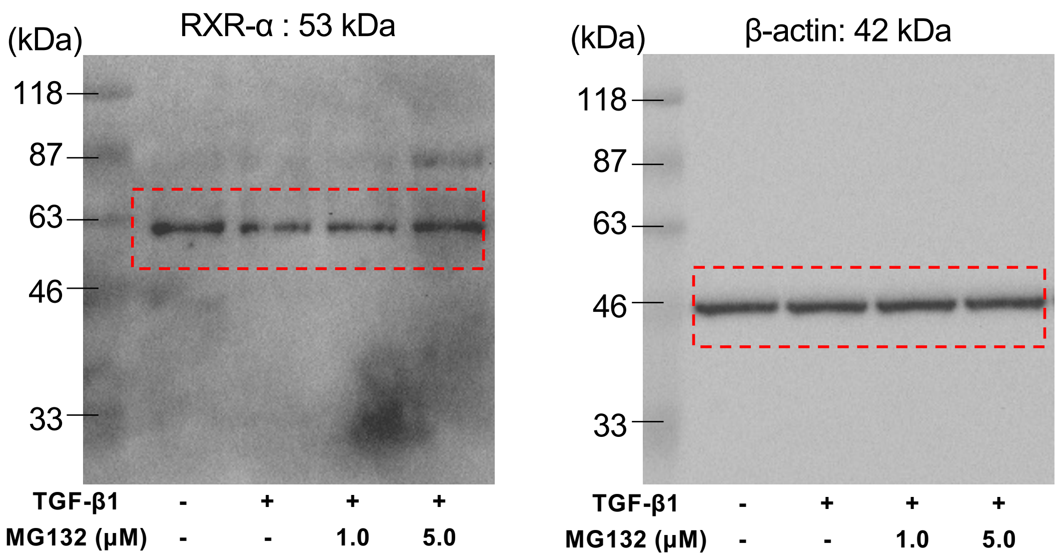

Figure.6B

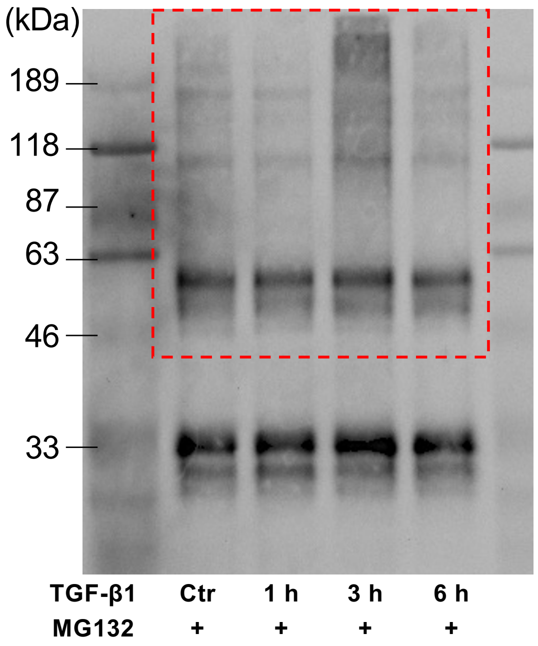

Image LabTM® 2.0 software (Bio-Rad, Hercules, CA, USA)

Figure.1A

rankl				
Avearage	1.051880452	7.850714061	3.138537438	4.277149277
S.D.	0.305465166	1.728971189	0.035524396	0.417398368
TGF-β1(ng/mL)	-	-	2.0	5.0
D_3+Dex	-	+	+	+

Figure.1B

opg				
Avearage	1.013265579	0.204928946	0.185475442	0.167127166
S.D.	0.183290206	0.052983014	0.020732254	0.023077951
TGF-β1(ng/mL)	-	-	2.0	5.0
D_3+Dex	-	+	+	+

Figure.1C

cyp24a1				
Avearage	1.051022511	107.4024266	0.588058081	43.98651063
S.D.	0.303472396	1.863015735	0.080432754	2.500640994
TGF-β1	-	-	+	+
D_3+Dex	-	+	-	+

Figure.1D

RANKL/β-actin			
Avearage	1	25.48321286	5.866344197
S.D.	0	0.714883128	0.522345625
TGF-β1	-	-	+
D_3+Dex	-	+	+

Figure.2B

TRAP positive multinucleated cells formed (cells/well)

Avearage	0	47.6666667	9
S.D.	0	2.05480467	1.63299316
TGF-β1	-	-	+
D_3+Dex	-	+	+

Figure.3A

rankl						
Avearage	1.02224638	7.07035983	3.89204002	5.53590761	5.210016742	7.61666986
S.D.	0.2146936	1.39747561	1.44823522	0.61329013	1.208157786	1.30686779
TGF-β1	-	-	+	+	+	+
D_3+Dex	-	+	+	+	+	+
A83-01(μM)	-	-	-	0.5	1.0	2.0

Figure.3B

RANKL/β-actin						
Avearage	1	11.22010851	4.313001552	9.522181715	9.548307734	8.218224615
S.D.	0	0.46457669	0.130517142	0.463134339	0.518568444	0.276855015
TGF-β1	-	-	+	+	+	+
D_3+Dex	-	+	+	+	+	+
A83-01(μM)	-	-	-	0.5	1.0	2.0

Figure.3C

smad2	si-control	si-smad2	si-smad3
Avearage	1.05957087	0.32561393	0.87673081
S.D.	0.33246056	0.09424545	0.26565279

smad3	si-control	si-smad2	si-smad3
Avearage	1.08463465	0.99612907	0.49373608
S.D.	0.09875975	0.1719714	0.15700655

Figure.3D

rankl(si-control)			
Avearage	1.00381095	3.43624203	1.47066174
S.D.	0.13313132	0.11665973	0.28727308

rankl(si-smad2)			
Avearage	1.03372602	5.39556516	4.96958987
S.D.	0.26121606	0.44725699	0.68776961

rankl(si-smad3)			
Avearage	1.14537076	10.5226306	5.22224373
S.D.	0.61533623	1.48444733	1.59743177
TGF-β1	-	-	+
D_3+Dex	-	+	+

Figure.4A

cytoplasmic fraction

VDR/β-actin				
Avearage	1	1.02231068	1.02984707	1.02074171
S.D.	0	0.01898764	0.02040898	0.00513268
TGF-β1	-	-	+	+
D_3+Dex	-	+	-	+

nuclear fraction

VDR/histone H3				
Avearage	1	2.55891079	1.46222165	3.09324265
S.D.	0	0.19371192	0.0192212	0.22004958
TGF-β1	-	-	+	+
D_3+Dex	-	+	-	+

Figure.4B

p-STAT3/STAT3				
Avearage	1	0.96935765	1.02016431	1.0773546
S.D.	0	0.0177188	0.03992582	0.01155526
TGF-β1	-	-	+	+
D_3+Dex	-	+	-	+

Figure.5A

RXR-α/β-actin					
Avearage	1	1.04178624	1.07564774	0.92791931	0.56924265
S.D.	0	0.08033257	0.05610592	0.04767121	0.00831385
TGF-β1	ctr	1h	3h	6h	12h

Figure.5B

rxr-α					
Avearage	1.01409576	1.64539673	1.11336971	1.29544453	1.32258769
S.D.	0.13606422	0.55579536	0.51150196	0.40894759	0.12456485
TGF-β1	ctr	1h	3h	6h	12h

Figure.5C

RXR-α/β-actin				
Avearage	1	0.51895933	1.32585877	1.15630516
S.D.	0	0.08342166	0.05157405	0.0798638
TGF-β1	-	+	-	+
A83-01	-	-	+	+

Figure.5D

RXR-α/β-actin				
	si-control		si-smad2/3	
Avearage	1	0.58366053	1	0.70285573
S.D.	0	0.05156133	0	0.00565418
TGF-β1	-	+	-	+

Figure.6A

RXR-α/β-actin				
Avearage	1	0.65902984	0.73099255	1.21631961
S.D.	0	0.04086315	0.01420581	0.08392873
TGF-β1	-	+	+	+
MG-132(μM)	-	-	1.0	5.0
