# Supplementary material for: Mechanisms involved in suppression of osteoclast supportive activity by transforming growth factor-β1 via the ubiquitin-proteasome system
Source: PLoS One. 2022 Feb 23;17(2):e0262612. doi: 10.1371/journal.pone.0262612 (PMC8865688; doi:10.1371/journal.pone.0262612)
Supplement: Attachment — Submitted filename: Comment.docx [file pone.0262612.s002.docx]

Comment 1) Effects of TGF-beta1 in vivo and in vitro.

In this study, the authors demonstrated TGF-beta 1 down-regulated RANKL expression in vitro.

TGF-beta1 is well known to exert suppressive effects in vitro, and to exert promotive effects in vivo.

Actually, the many manuscript has been published already regarding TGF-beta 1 derived from bone matrix promoted osteoclastgenesis.

Therefore, this study demonstrated one-sided effects of TGF-beta 1 and effects of TGF-beta 1 is already known to the potential readers.

The authros should revise the manuscript extensively so that the potential readers can find new findings, and recognize this study does not contain one-sided effects.

Comment 2) Stromal cells or mesenchymal stem cells ?

In general, stromal cells involves all the non-epithelial cells including fibroblast, lipocytes, lymphcytes, muscular cells, and neuron cells. Only in the field of regeneration, a stromal cell is referred to as a mesenchymal stem cell (MSC). To avoid misunderstanding of the potential readers, the stromal cells in the current study should be revised as mesenchymal stem cells.

Comment 3) Many sentences involving unclear words and meanings

There are many many sentences involving unclear words and meanings. These sentences should be revised before submission. The following are the examples.

Example 1) The current study investigated the effect of TGF-β1 on receptor activator of nuclear factor kappa-B ligand (RANKL) expression, induced by 1α,25(OH)2D3 (D3) and dexamethasone (Dex) in stromal cells.

This is very complex. RANKL expression in stromal cell

Example 2) Stromal cells support osteoclast genesis. ; Please explain the meaning of "support osteoclastgenesis” in this study. Is this mean "induce osteoclastgenesis"? What is the difference?

Example 3) ST2 cells in the abstract section. Please explain what ST2 cells mean in the abstract section.

Example 4) Transforming growth factor-β1 (TGF-β1) is a coupling factor vital for bone remodeling.

What means "vital bone remodeling" ?
