# Supplementary material for: Mechanisms involved in suppression of osteoclast supportive activity by transforming growth factor-β1 via the ubiquitin-proteasome system
Source: PLoS One. 2022 Feb 23;17(2):e0262612. doi: 10.1371/journal.pone.0262612 (PMC8865688; doi:10.1371/journal.pone.0262612)
Supplement: Attachment — Submitted filename: Response to Reviewers.docx [file pone.0262612.s003.docx]

**Answer to the academic editor’s comments**

Comment 1) Effects of TGF-β1 *in vivo* and *in vitro.*

In this study, the authors demonstrated TGF-β1 down-regulated RANKL expression *in vitro*. TGF-β1 is well known to exert suppressive effects *in vitro*, and to exert promotive effects *in vivo*. Actually, the many manuscripts have been published already regarding TGF-β1 derived from bone matrix promoted osteoclastgenesis. Therefore, this study demonstrated one-sided effects of TGF-beta 1 and effects of TGF-β1 is already known to the potential readers. The authors should revise the manuscript extensively so that the potential readers can find new findings, and recognize this study does not contain one-sided effects.

We appreciate your important suggestions. Several studies demonstrated that the role of TGF-β1 on bone remodeling process. However, the reported effects of TGF-β1 are controversial. Since many of these studies are focused on the direct effects of TGF-β1 on osteoclast progenitors, we investigated the effects of TGF-β1 on RANKL generation in stromal cells. Moreover, the role of TGF-β1 as a coupling factor of bone remodeling process should be elucidated. However, we did not mention about it. We edited the Discussion session in the revised manuscript with some references.

Comment 2) Stromal cells or mesenchymal stem cells?

In general, stromal cells involves all the non-epithelial cells including fibroblast, lipocytes, lymphcytes, muscular cells, and neuron cells. Only in the field of regeneration, a stromal cell is referred to as a mesenchymal stem cell (MSC). To avoid misunderstanding of the potential readers, the stromal cells in the current study should be revised as mesenchymal stem cells.

We appreciate your helpful suggestions. ST2 cell is a cloned stromal cell line from mouse bone marrow. This cell line has the ability to differentiate into osteoblast-like cells and shows high expression of RANKL and low expression of OPG when treated with 1,25(OH)_2_D_3_ and dexamethasone. However, we did not mention about it. So, we added this information in the Materials and methods section of revised manuscript.

Comment 3) Many sentences involving unclear words and meanings.

There are many sentences involving unclear words and meanings. These sentences should be revised before submission. The following are the examples.

We appreciate your helpful comments. We carefully checked and revised the sentences that were pointed out.

Example 1) The current study investigated the effect of TGF-β1 on receptor activator of nuclear factor kappa-B ligand (RANKL) expression, induced by 1α,25(OH)_2_D_3_ (D3) and dexamethasone (Dex) in stromal cells. This is very complex. RANKL expression in stromal cell

Thank you for your helpful comments. We edited the abstract section of revised manuscript.

Example 2) Stromal cells support osteoclastogenesis.; Please explain the meaning of "support osteoclastogenesis” in this study. Is this mean "induce osteoclastogenesis"? What is the difference?

Thank you for your suggestions. Previous studies have been revealed the direct effect of TGF-β1 on the osteoclast differentiation from osteoclast progenitor cells. In this study, we showed that TGF-β1 indirectly inhibits the expression of RANKL, an accelerator of osteoclast differentiation in stromal cells. To emphasize these differences, we used the term “support osteoclastogeneis” in the place of “induce osteoclastogenesis”.

Example 3) ST2 cells in the abstract section. Please explain what ST2 cells mean in the abstract section.

As noted by the reviewer’s comment, we added the information of ST2 cells in the abstract section.

Example 4) Transforming growth factor-β1 (TGF-β1) is a coupling factor vital for bone remodeling. What means "vital bone remodeling"?

As noted by the reviewer’s comment, we edited the abstract section of revised manuscript.
